# Establishment and validation of a risk model for prediction of in-hospital mortality in patients with acute ST-elevation myocardial infarction after primary PCI

**DOI:** 10.1186/s12872-020-01804-7

**Published:** 2020-12-09

**Authors:** Nan Gao, Xiaoyong Qi, Yi Dang, Yingxiao Li, Gang Wang, Xiao Liu, Ning Zhu, Jinguo Fu

**Affiliations:** 1 Department of Internal Medicine, Hebei Medical University, Shijiazhuang, Hebei China; 2 Department of Cardiology, Hebei General Hospital, Shijiazhuang, Hebei China; 3 Department of Cardiology, Cangzhou Central Hospital, Cangzhou, Hebei China

**Keywords:** Predictive value of tests, Nomogram, ST-elevated myocardial infarction, Percutaneous coronary intervention, Hospital mortality

## Abstract

**Background:**

Currently, how to accurately determine the patient prognosis after a percutaneous coronary intervention (PCI) remains unclear and may vary among populations, hospitals, and datasets. The aim of this study was to establish a prediction model of in-hospital mortality risk after primary PCI in patients with acute ST-elevated myocardial infarction (STEMI).

**Methods:**

This was a multicenter, observational study of patients with acute STEMI who underwent primary PCI. The outcome was in-hospital mortality. The least absolute shrinkage and selection operator (LASSO) method was used to select the features that were the most significantly associated with the outcome. A regression model was built using the selected variables to select the significant predictors of mortality. Receiver operating characteristic (ROC) curve and decision curve analysis (DCA) were used to evaluate the performance of the nomogram.

**Results:**

Totally, 1169 and 316 patients were enrolled in the training and validation sets, respectively. Fourteen predictors were identified by the LASSO analysis: sex, Killip classification, left main coronary artery disease (LMCAD), grading of thrombus, TIMI classification, slow flow, application of IABP, administration of β-blocker, ACEI/ARB, symptom-to-door time (SDT), symptom-to-balloon time (SBT), syntax score, left ventricular ejection fraction (LVEF), and CK-MB peak. The mortality risk prediction nomogram achieved good discrimination for in-hospital mortality (training set: C-statistic = 0.987; model calibration: *P* = 0.722; validation set: C-statistic = 0.984, model calibration: *P* = 0.669). Area under the curve (AUC) values for the training and validation sets are 0.987 (95% CI: 0.981–0.994, *P* = 0.003) and 0.990 (95% CI: 0.987–0.998, *P* = 0.007), respectively. DCA shows that the nomogram can achieve good net benefit.

**Conclusions:**

A novel nomogram was developed and is a simple and accurate tool for predicting the risk of in-hospital mortality in patients with acute STEMI who underwent primary PCI.

## Background

Acute myocardial infarction (AMI) is a leading cause of death worldwide [1–3]. In the United States, the incidence of AMI was 208 per 100,000 person-years in 2008 [4]. In patients with symptoms of myocardial ischemia, ST-elevation myocardial infarction (STEMI) is defined by the combination of persistent ST-segment elevation and the subsequent release of biomarkers of myocardial necrosis [5]. Other types of acute coronary syndromes include non-ST-elevation myocardial infarction (NSTEMI) and unstable angina (UA) [5]. STEMI is most often caused by the rupture of an atherosclerotic plaque in the culprit coronary artery, followed by total occlusion with a thrombus [6, 7]. Common risk factors for AMI include tobacco abuse, dyslipidemias, hypertension, diabetes mellitus, and family history of coronary artery diseases (CAD) [8, 9].

Primary PCI is one of the first-line therapeutic strategies for acute STEMI, and increasing evidence suggests that primary PCI can improve the prognosis of AMI [10–13]. Nevertheless, the mortality risk is still high, especially in patients with AMI complicated by cardiogenic shock and malignant arrhythmia, despite the use of other management modalities such as intra-aortic balloon pump (IABP), percutaneous cardiopulmonary support (PCPs), and other mechanical auxiliary devices [14–16].

Various models based on clinical and angiographic variables are available for determining the prognosis of AMI after PCI [17–20]. Data from the New York PCI Reporting System allowed the creation of two scoring systems for the determination of the in-hospital and 30-day prognosis after PCI [21, 22]. Recently, a deep-learning machine analysis was used to create a nomogram for in-hospital mortality, in which age and ejection fraction were the major predictors [16]. Currently, how to accurately determine the prognosis of the patients after PCI remains unclear and may vary among populations, hospitals, and datasets [23, 24]. Those models remain imperfect, and many patients are incorrectly classified, which can have an impact on the aggressiveness of their management and their prognosis.

Therefore, the aim of the present study was to establish a prediction model of in-hospital mortality risk by analyzing the data of patients with acute STEMI who underwent primary PCI. The results could help the clinicians determine the early diagnosis and identify high-risk patients.

## Methods

### Study design and patients

This was a multicenter, observational study of the data of patients treated between January 2016 and December 2018 at the Hebei General Hospital, Baoding First Central Hospital, and Cangzhou Central Hospital. All patients were meeting the diagnostic criteria of acute STEMI and underwent primary PCI according to current guidelines [25]. The exclusion criteria were: (1) STEMI but no primary PCI; or (2) acute non-STEMI or unstable angina.

The patients who were retrospectively included constituted the training set (January 2016 to June 2018). The validation set contained patients who were prospectively enrolled (July 2018 to December 2018) according to the same criteria so as to avoid modeling bias caused by similar populations. The study was approved by the ethics committees of Hebei General Hospital, Baoding First Central Hospital, and Cangzhou Central Hospital. The patients and their immediate family members consented to receive a primary intervention. The written consent was obtained from study participants and their immediate family.

### Data collection

General data (age, sex, body mass index (BMI), smoking, and alcohol consumption), past medical history (including coronary heart disease, history of angina pectoris, hypertension, type 2 diabetes mellitus, myocardial infarction, cerebral infarction, chronic kidney disease, coronary intervention, atrial fibrillation, and cerebral hemorrhage), vital signs at admission (body temperature, pulse, respiratory rate, systolic blood pressure, diastolic blood pressure, Killip classification, and location of the myocardial infarction), auxiliary examinations (white blood cell count, neutrophil count, eosinophil count, basophil count, red blood cell count, hemoglobin, platelet count, serum potassium, serum sodium, serum chlorine, alanine aminotransferase (ALT), aspartate aminotransferase (AST), serum creatinine, uric acid, cholesterol, triglycerides (TG), low-density lipoprotein (LDL-C), high-density lipoprotein (HDL-C), very low-density lipoprotein (VLDL-C), random blood glucose, creatine kinase MB (CK-MB) peak, and left ventricular ejection fraction (EF)), chest pain data, interventions (culprit vessels, location, diameter, length, vessel number of lesions, treatment of non-culprit vessels or not, preoperative TIMI flow, presence or absence of collateral circulation, syntax score, grading of thrombus, thrombus aspiration, number of, grading of postoperative TIMI flow, IABP, intraoperative slow flow, intraoperative ventricular tachycardia, intraoperative ventricular fibrillation, and intraoperative cardiac tamponade), stents (most of the stents used in this study were EXCEL drug-coated stents (Jiwei Medical Products Co., Ltd.) and all other stents were drug-coated stents), medications (administration of β-blocker, angiotensin converting enzyme inhibitor/angiotensin receptor blocker (ACEI/ARB), aldosterone, diuretic, nicorandil, and calcium channel blocker or not 3 months before admission), and administration of the above medications after admission were collected from the medical charts.

### Definitions

In-hospital mortality was defined as all-cause mortality during hospitalization. The assessment of the left ventricular function by transthoracic echocardiography after STEMI was confirmed. The patient was in the supine position, and according to the frontier approaches of the American Society of Echocardiography, at least three consecutive cardiac cycles were used to measure the internal dimensions of the left ventricle (i.e., the end-systolic diameter and the end-diastolic diameter). LVEF was calculated as follows:

$${\text{LVEF }}\left( \% \right) \, = \, \left[ {\left( {{\text{LVEDD}}^{{3}} - {\text{LVEDS}}^{{3}} } \right)/{\text{LVEDD}}^{{3}} } \right] \, \times { 1}00\% .$$

Chest pain data included symptom-to-door time, symptom-to-antiplatelet administration time, symptom-to-anticoagulant administration time, symptom-to-balloon time, first medical contact-to-antiplatelet administration time, first medical contact-to-anticoagulant administration time, and first medical contact-to-balloon time. The time of chest pain onset was determined by asking the patient to and consulting the family accompanying the patient. After admission, ECG and blood sampling were done within 10 min. According to the presence or not of a Q wave, the dynamic evolution of ST-T, and whether the blood myoglobin and CK-MB levels were elevated, self-reported chest pain onset time was validated. The balloon expansion time was determined based on Chinese chest pain data provided by the center.

Killip class I included the patients with no clinical signs of heart failure. Killip class II included the patients with AMI complicated by left heart failure, with moist rales of both lungs being less than 50% of the lung field. Killip class III included the patients with AMI complicated with acute pulmonary edema, with large, small, dry, and moist rales of the whole lung. Killip class IV included the patients with AMI complicated with hemodynamic changes at different degrees or stages, such as cardiogenic shock [26].

TIMI 0 flow (no perfusion) referred to the absence of any antegrade flow beyond the coronary occlusion. TIMI 1 flow (penetration without perfusion) referred to faint antegrade coronary flow beyond the occlusion, with an incomplete filling of the distal coronary bed. TIMI 2 flow (partial reperfusion) referred to delayed or sluggish antegrade flow with complete filling of the distal territory. TIMI 3 flow was a normal flow that filled the distal coronary bed completely [27].

The thrombus score was assessed after the guidewire passed through the lesion (but before balloon dilatation). A significant filling defect in the lumen could be seen, which was visible in multiple angles of angiography and persistently present over multiple cardiac cycles, and after excluding the interlayer of the inner membrane caused by the guidewire in the false lumen. The thrombus score was graded as 0: no thrombus; 1: haziness; 2: definite thrombus < 1/2 vessel diameter; 3: definite thrombus 1/2 to 2 vessel diameters; 4: definite thrombus > 2 vessel diameters; 5: assessing thrombus was impossible due to vascular occlusion [28].

### Selection of predictors

The least absolute shrinkage and selection operator (LASSO) method was used to select the features that were the most significantly associated with the outcome (in-hospital mortality). Then, a regression model was built using the selected variables [29]. Originally proposed for linear regression models, this method minimizes the residual sum of squares, subject to the sum of the absolute value of the coefficients being less than a tuning parameter (λ). For the binary logistic regression model, the residual sum of squares was replaced by the negative log-likelihood. If λ was large, there was no effect on the estimated regression parameters, but as λ was smaller, some coefficients were shrunk to zero [30, 31]. Then, the λ value was selected for which the cross-validation error was the smallest. Finally, the model was re-fitted using all available observations and the selected λ. Thus, most of the coefficients of the covariates were reduced to zero, and the remaining non-zero coefficients were selected by LASSO. The variable factor of a non-zero coefficient was defined as a mortality risk predictor. Therefore, in the present study, the mortality risk score for each patient was calculated by a linear combination of predictors that were weighted by their respective coefficients. The performance of the nomogram was evaluated in terms of discrimination and calibration. Discrimination was quantified using the area under the receiver operating characteristic (ROC) curve. The extent of over- and underestimation was graphically described using calibration plots. Decision curve analysis (DCA) was used to evaluate the net benefit of the model [32–34].

### Statistical analysis

Statistical analysis was performed using R version 3.3.0 (R Foundation for Statistical Computing). All data were normalized by transforming the data into new scores (z-score transformation) with a mean of 0 and a standard deviation of 1. The glmnet R package was used for the LASSO regression model. The mortality risk score for each patient was calculated as a linear combination of selected predictors that were weighted by their respective coefficients. The “rms” package was used for the mortality risk prediction nomogram. The predictive accuracy of the risk model was assessed by discrimination, measured using the C-statistic, and calibration, evaluated by the Hosmer–Lemeshow chi-square statistic. The differences in various variables between the mortality and surviving groups were assessed by using an independent samples t-test, chi-square test, or Mann–Whitney U-test, as appropriate. The normality test was conducted using the Kolmogorov–Smirnov test. The continuous variables with a normal distribution were presented as the mean ± standard deviation, and those with a non-normal distribution were presented as the median (interquartile range). The categorical variables were expressed as n (%). All statistical tests were two-sided, with a *p* value < 0.05 being considered significant.

## Results

### Characteristics of the patients

A total of 1485 acute STEMI patients who underwent primary PCI were included in this study, including 1169 in the training set (95 (8.1%) dead patients and 1074 (91.9%) survivors) and 316 (25 (7.9%) dead patients and 291 (92.1%) survivors) in the validation set. The clinical characteristics of the patients in the two sets are shown in Table 1. The proportions of males were 74.9% and 71.2% in the training and validation sets, respectively, for a ratio of 1.05 between the two sets. The hospital stay was 10.9 ± 3.6 days in the training set and 10.3 ± 3.3 days in the validation set (*P* = 0.394).

**Table 1:** Clinical characteristics of the patients in the training and validation sets used to construct the nomogram, according to the in-hospital mortality status

Clinical characteristics	Training set (n = 1169)			Validation set (n = 316)		
	In-hospital mortality (n = 95)	Survival (n = 1074)	*P*	In-hospital mortality (n = 25)	Survival (n = 291)	*P*
Male	60 (63.2)	816 (76.0)	0.006*	18 (72.0)	207 (71.1)	0.927
Age (years)	66.3 ± 13.3	59.6 ± 11.4	< 0.001*	66.0 ± 14.2	60.0 ± 12.1	0.298
BMI (kg/m^2^)	25.0 ± 3.9	25.5 ± 3.3	0.011*	25.5 ± 4.0	25.4 ± 3.4	0.077
Drinking history	22 (23.2)	297 (27.7)	0.346	8 (32.0)	68 (23.4)	0.332
Smoking history	35 (36.8)	518 (48.2)	0.033*	8 (32.0)	121 (41.6)	0.350
DM history	26 (27.4)	214 (19.9)	0.085	7 (28.0)	59 (20.3)	0.362
Hypertension history	51 (53.7)	516 (48.0)	0.292	10 (40.0)	139 (47.8)	0.455
Killip classification			< 0.001*			< 0.001*
I	35 (36.8)	939 (87.4)		12 (48.0)	262 (90.0)	
II	14 (14.7)	110 (10.2)		5 (20.0)	21 (7.2)	
III	8 (8.4)	18 (1.7)		1 (4.0)	6 (2.1)	
IV	38 (40.0)	7 (0.7)		7 (28.0)	2 (0.7)	
LMCAD	7 (7.4)	3 (0.3)	< 0.001*	2 (8.0)	1 (0.3)	< 0.001*
Grading of thrombus			0.005*			0.340
0	0	6 (0.6)		0	2 (0.7)	
1	0	15 (1.4)		3 (12.0)	5 (1.7)	
2	2 (2.1)	96 (8.9)		3 (12.0)	32 (11.0)	
3	29 (30.5)	450 (41.9)		6 (24.0)	131 (45.0)	
4	44 (46.3)	348 (32.4)		10 (40.0)	94 (32.3)	
5	20 (21.1)	159 (14.8)		3 (12.0)	42 (14.4)	
TIMI classification			< 0.001*			< 0.001*
0	16 (16.8)	1 (0.1)		6 (24.0)	0	
1	10 (10.2)	2 (0.2)		3 (12.0)	1 (0.3)	
2	12 (12.6)	57 (5.3)		2 (8.0)	15 (5.1)	
3	57 (60.0)	1014 (94.4)		14 (56.0)	275 (94.5)	
Slow flow	41 (43.2)	86 (8.0)	< 0.001*	7 (28.0)	25 (8.6)	< 0.001*
Application of IABP	18 (19.0)	14 (1.3)	< 0.001*	4 (16.0)	3 (1.0)	< 0.001*
Administration of β-blocker	32 (33.7)	809 (75.3)	< 0.001*	6 (24.0)	236 (81.1)	< 0.001*
ACEI/ARB	20 (21.1)	644 (60.0)	< 0.001*	5 (20.0)	186 (63.9)	0.003*
Symptom-to-door time (min)	256 ± 235	89 ± 73	< 0.001*	248 ± 226	85 ± 74	< 0.001*
Symptom-to-balloon time (min)	426 ± 244	236 ± 153	< 0.001*	420 ± 269	234 ± 157	< 0.001*
Syntax score	29.3 ± 9.9	20.7 ± 7.7	0.003*	31.9 ± 13.1	20.9 ± 7.9	0.003*
EF (%)	47.2 ± 8.5	54.6 ± 8.1	0.918	45.1 ± 7.4	54.7 ± 7.64	0.926
CK-MB (U/L)	180.0 ± 157.2	156.4 ± 58.2	< 0.001*	175.3 ± 197.5	158.9 ± 54.7	0.019*
Random blood glucose (mmol/L)	9.25 ± 3.51	8.06 ± 2.83	0.056	9.3 (7.4,15.71)	7.1 (6.19,9.28)	0.001*
Triglycerides (mmol/L)	1.42 (1.01,1.80)	1.43 (0.99,1.99)	0.687	1.28 (0.98,1.71)	1.49 (0.99,1.99)	0.290

*P < 0.05 between the in-hospital mortality and survival groups

*BMI* body mass index, *DM* diabetes mellitus, *LMCAD* left main coronary artery disease, *TIMI* thrombolysis in myocardial infarction, *IABP* intra-aortic balloon pump, *ACEI* angiotensin-converting enzyme inhibitor, *ARB* angiotensin receptor blocker, *EF* ejection fraction, *CK-MB* creatinine kinase MB

### Selection mortality risk predictors and development of a risk prediction model

**Fig. 1:**
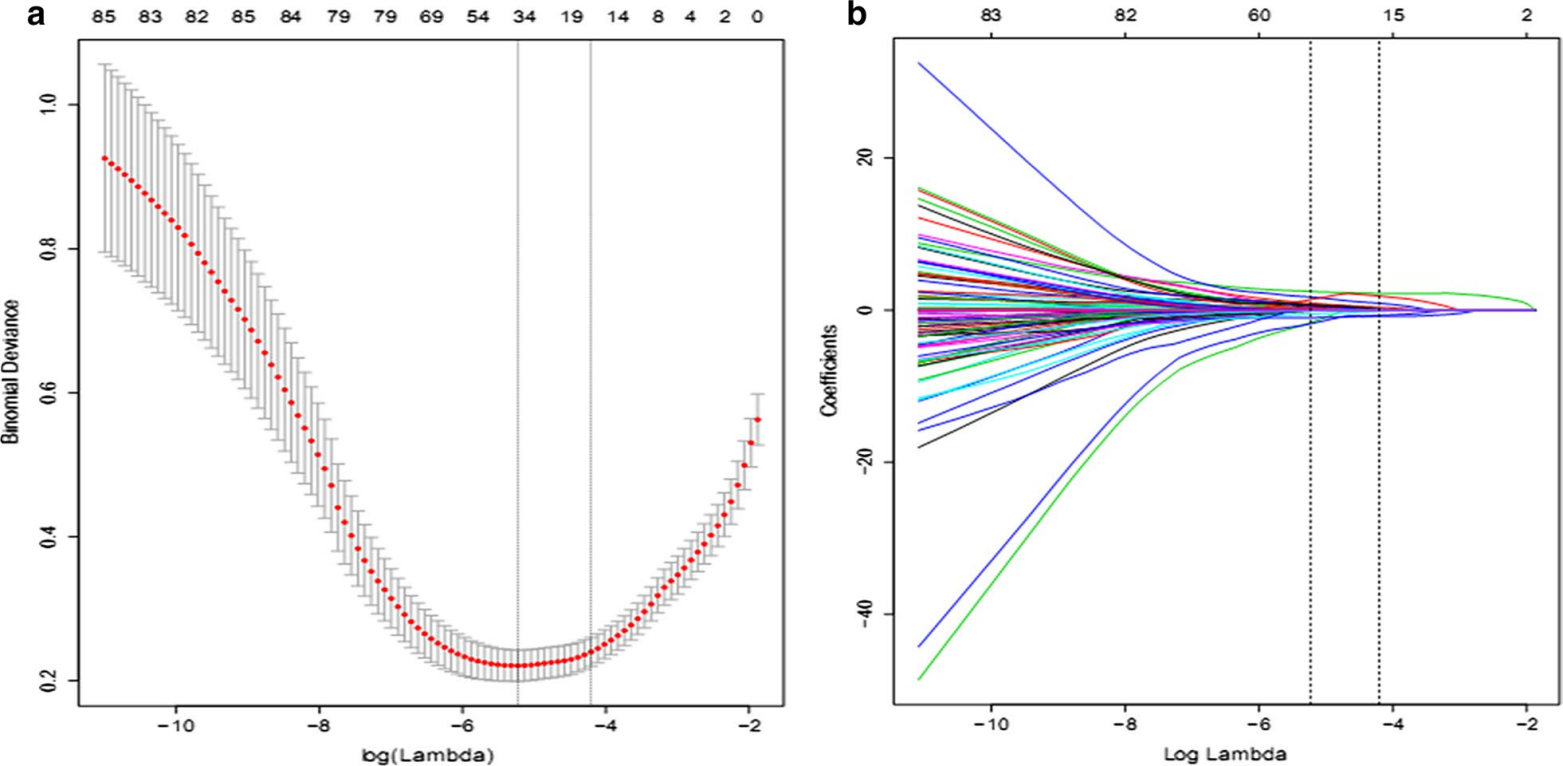
Texture feature selection using the least absolute shrinkage and selection operator (LASSO) binary logistic regression model. **a** The tuning parameter (λ) selection in the LASSO model used tenfold cross-validation via minimum criteria. The area under the receiver operating characteristic (AUC) curve was plotted versus log(λ). Dotted vertical lines were drawn at the optimal values by using the minimum criteria and the 1 standard error of the minimum criteria. The λ value was 0.003. **b** LASSO coefficient profiles of the 81 features. A coefficient profile plot was produced against the log(λ) sequence. Vertical lines were drawn at the value selected using tenfold cross-validation, where optimal λ resulted in 14 non-zero coefficients

Based on the LASSO analysis, sex, Killip classification, LMCAD, thrombus score, postoperative TIMI flow grading, intraoperative slow flow, IABP, administration of β-blocker, ACEI/ARB, SDT, SBT, syntax score, EF, and CK-MB peak were selected as predictors for the development of the mortality risk prediction model (Fig. 1a,b).

### Mortality risk calculation

**Fig. 2:**
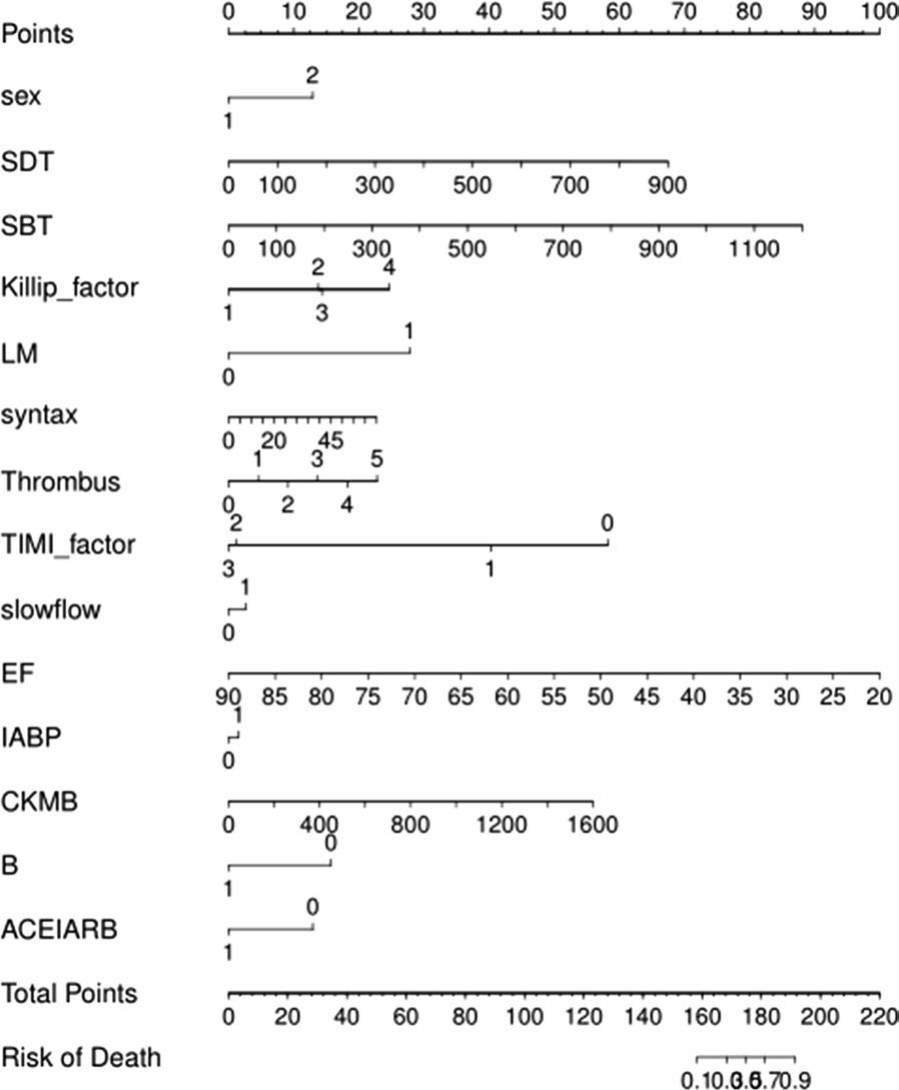
The mortality risk prediction nomogram. SDT: symptom-to-door time; SBT: symptom-to-balloon time; LMCAD: left main coronary artery disease; TIMI: thrombolysis in myocardial infarction; IABP: intra-aortic balloon pump; ACEI: angiotensin-converting enzyme inhibitor; ARB: angiotensin receptor blocker; EF: ejection fraction; CK-MB: creatinine kinase MB; B: β-blocker

The mortality risk score was calculated as follows:

$$\begin{aligned} & {\text{mortality}}\;{\text{risk}}\;{\text{score }} = { 2}.{61871} + {1}.{5829}0{5 } \times {\text{ sex}} + 0.0{1}0{47}0{1 } \times {\text{ SDT}} + 0.00{85}0{25 } \\ & \quad \times {\text{ SBT}} + {1}.00{8971 } \times {\text{ Killip}} + {3}.{274}0{61 } \times {\text{ LMCAD }} + 0.0{493}0{55 } \times {\text{ syntax}} + 0.{6}0{38351} \\ & \quad \times {\text{ thrombus - 2}}.{647184 } \times {\text{ TIMI - 1}}.{1558}0{4 } \times {\text{ slowflow}} + 0.{2368332 } \times {\text{ IABP - }}0.{174}0{645} \\ & \quad \times {\text{ EF}} + 0.00{47845 } \times {\text{ CKMB}} - {1}.{973}0{87 } \times \, \beta {\text{ - blocker - 1}}.{5187}0{2 } \times {\text{ ACEI}}/{\text{ARB}}, \\ \end{aligned}$$

in which male sex was scored as 1 and female sex as 2; for LMCAD, intraoperative slow flow, IABP, administration of β-blocker, and ACEI/ARB, yes was scored as 1, no as 0; Killip classification was scored as 1–4; the thrombus score was scored as 0–5; postoperative TIMI flow grading was scored as 0–3; and the remaining variables were continuous variables. This equation was used to design a nomogram (Fig. 2).

### Validation of the prediction model

**Fig. 3:**
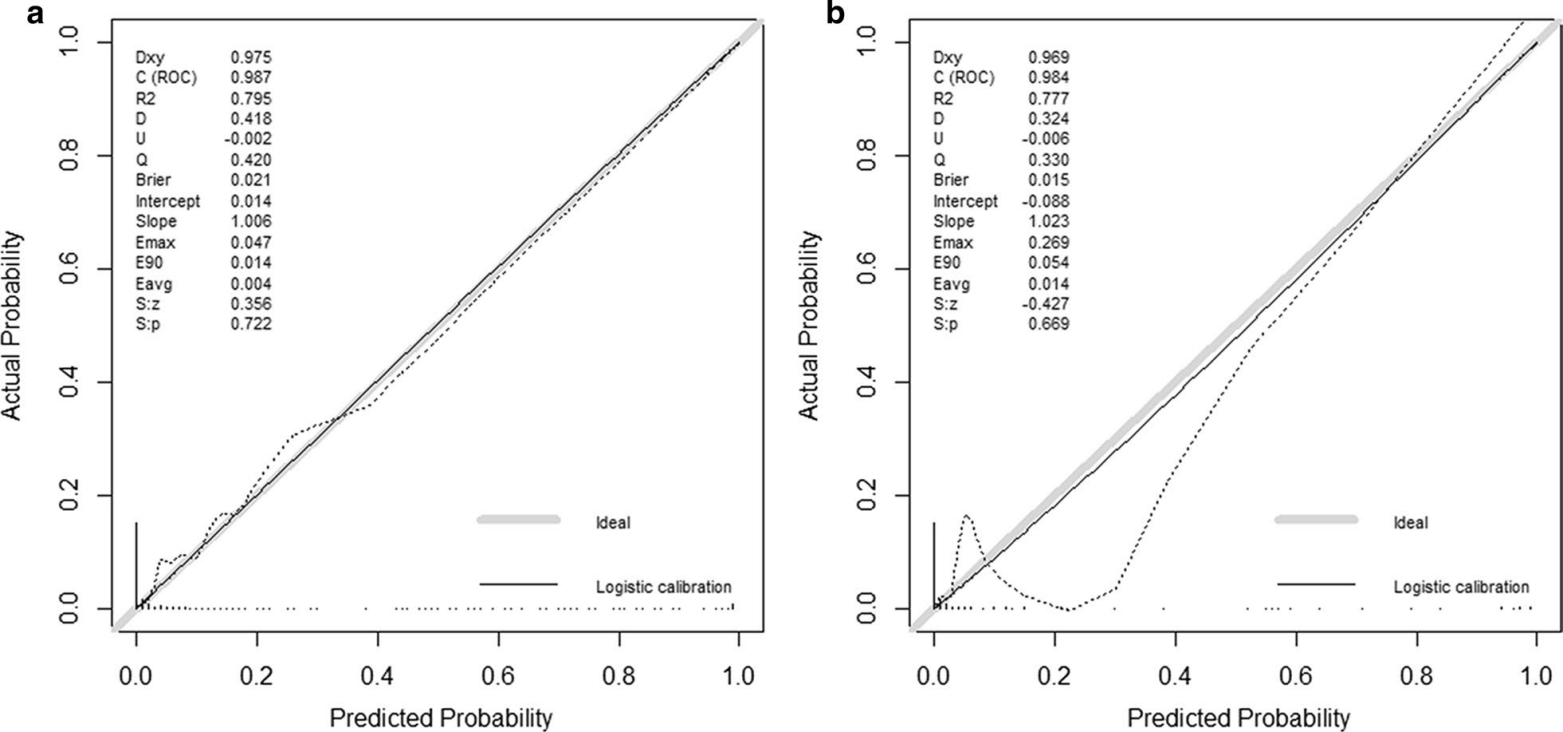
**a** Validation of the prediction model in the training set **b** validation of the prediction model in the validation set

**Fig. 4:**
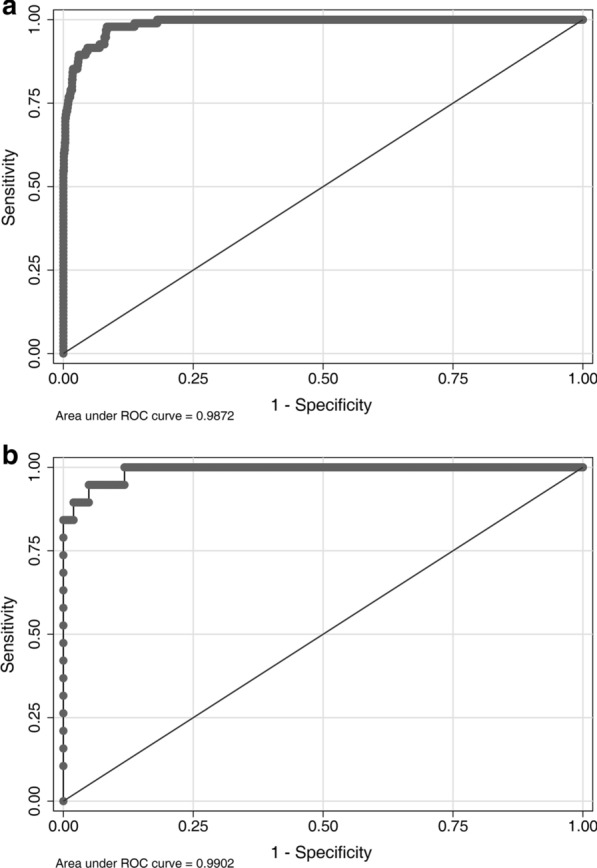
**a** Receiver operating characteristic (ROC) curve of the nomogram in the training set (area under the curve (AUC) = 0.987, 95% confidence interval (CI): 0.981–0.994, *P* = 0.003). Sensitivity was 97.9%, specificity was 91.6%. **b** ROC curve of the nomogram in the validation set (AUC = 0.990, 95% CI: 0.987–0.998, *P* = 0.007). Sensitivity was 94.7%, specificity was 95.1%

**Fig. 5:**
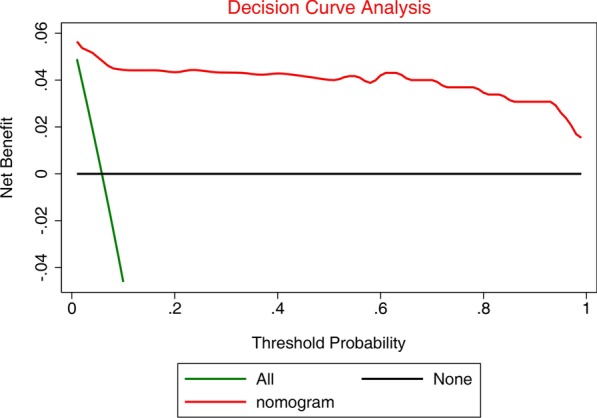
Decision curve analysis (DCA) shows that the nomogram can achieve good net benefit

The prediction model was validated in the training and validation sets. The C-statistic in the training set was 0.987 (Fig. 3a), indicating that the nomogram had good discrimination. The prediction accuracy of the nomogram was evaluated by the Hosmer–Lemeshow chi-square statistic calibration method and revealed *P* = 0.722, indicating that the nomogram had good prediction accuracy. The C-statistic in the validation set was 0.984 (Fig. 3b), indicating that the nomogram had good discrimination. The prediction accuracy of the nomogram revealed *P* = 0.669, indicating that the nomogram had good prediction accuracy. Figure 4a shows the ROC curve in the training set (AUC = 0.987, 95% confidence interval (CI): 0.981–0.994, *P* = 0.003). Sensitivity was 97.9%, specificity was 91.6%. Figure 4b shows the ROC curve in the validation set (AUC = 0.990, 95% CI: 0.987–0.998, *P* = 0.007). Sensitivity was 94.7%, specificity was 95.1%. Figure 5 shows the DCA of the prediction nomogram. DCA reveals that the nomogram can achieve good net benefit.

### Use of the nomogram

The supplementary materials present an example of how to calculate the score using the nomogram (Additional file 1).

### Diabetes

Table 2 shows that the proportions of patients with random blood glucose levels > 10 mmol/L was 29.5% in the mortality group and 22.2% in the survival group (*P* = 0.103). In addition, among 240 patients with diabetes, there were no significant differences in the proportions of those treated by diet management alone, oral hypoglycemic drugs, and insulin with or without oral hypoglycemic drugs (*P* = 0.813).

**Table 2:** Analysis based on diabetes

	Death (n = 95)	Survival (n = 1074)	*P*
Random blood glucose (mmol/L)			
≤ 10	67 (70.5%)	836 (77.8%)	0.103
> 10	28 (29.5%)	238 (22.2%)	
Treatments			
Diet management	2 (8.7%)	14 (6.5%)	0.813
Oral hypoglycemic drugs	14 (60.9%)	124 (57.1%)	
Insulin with or without oral hypoglycemic drugs	7 (30.4%)	79 (36.4%)	

## Discussion

This study found that the risk of in-hospital mortality in women with AMI undergoing primary PCI was higher than that in men, which was similar to the results of clinical observations by Tsai et al. [12], Stehli et al. [35], and Guo et al. [36]. This might be because of the atypical symptoms of chest pain in women, leading to a longer time from symptom-to-first medical contact than in men.

Regarding the first medical contact time, prior studies focused more on the door-to-balloon time and believed that reducing the door-to-balloon time could improve the prognosis of patients with AMI [37]. On the other hand, Prasad et al. [38] showed that for patients with STEMI, the delayed mechanical opening of the infarct-related artery was associated with damage to the microcirculation and that the symptom-to-balloon time was more significant than door-to-balloon time for this correlation. In previous studies, reducing the door-to-balloon time was beneficial for myocardial perfusion [37], but not in patients with duration of symptoms > 120 min [39], indicating that a reduction in the door-to-balloon time does not result in a reduction in mortality in patients with STEMI [40, 41]. The present study found that with the reduction in door-to-balloon time, the mortality risk was reduced, which is similar to the findings of Dudek et al. [42].

Recently, Kim et al. [43] confirmed that even in patients with acute STEMI after primary PCI, 14% still had left ventricular dysfunction, with CK-MB being an independent predictor for the decrease in LVEF. The present study found that patients with a high CK-MB peak had a higher mortality risk than those with a low one. LVEF and TIMI grade were also included in the nomogram.

The present study is not the first to try to determine the prognosis of AMI after PCI [17–20]. Indeed, the SYNTAX and derived scores have been shown to predict the prognosis after PCI [19]. Weintraub et al. [20] proposed a 24-variable model that has a C-value of 0.79 in STEMI prognosis after PCI. Hannan et al. [21] proposed an 11-item score that has a high predictive value. A recent deep-learning machine analysis was used to create a nomogram for in-hospital mortality, in which age and ejection fraction were the major predictors [16]. A recent review presented the main models used for the prognosis of STEMI [44]. Taken together, those models and the present one globally used the same variables but with different weighted values. In addition, they were obtained in different populations, impairing a direct comparison among the studies.

As indicated in these studies [45–51], diabetes and mainly hyperglycemia have been both investigated as a risk factor for in-hospital and long-term mortality in patients treated with primary PCI for STEMI. Indeed, as suggested by Kogan et al. [45], Abizaid et al. [46], and Marui et al. [47], we could see that diabetes was not associated with postoperative in-hospital mortality. On the other hand, as indicated by Kogan et al. [45], Marfella et al. [48], Sardu et al. [49], and D’Onofrio et al. [50] diabetes, and mainly hyperglycemia, could affect in-hospital and long-term mortality in patients hospitalized for STEMI. In the present study, 29.5% of the patients in the mortality group and 22.2% of those in the survival group had random blood glucose levels > 10 mmol/L (*P* = 0.103), and there were no differences in the diabetes treatments either (*P* = 0.813).

The clinical implication of such predictive models is to improve the management of patients through personalized medicine [5, 8, 17, 24, 25, 40, 41]. Indeed, overtreatment will lead to unnecessary healthcare expenses and the exposure of the patient to adverse effects of drugs or unnecessary interventions, tipping the risk/benefit balance towards the risks. On the other hand, undertreatment will also tip the risk/benefit balance toward additional risks, but this time from the primary condition that can recur or complicate because of insufficient treatment. The previous models have several disadvantages and do not allow for a proper personalization of treatments [16–24]. Nevertheless, how the model determined in the present study can be used to personalize the treatments remains to be explored. Of note, the model in the present study had high AUC in both cohorts, and the DCA showed that it could achieve a good net benefit.

This study has several limitations. First, it was a retrospective observational study, not a randomized, controlled clinical study. Although the selection of the patients was performed in a multicenter manner, selection bias is inevitable. Given it was a multicenter observational study with more patients than in previous studies, the results may be more reliable. Nevertheless, the nomogram proposed here was not directly compared with other available models. Second, LASSO is generally considered superior to logistic regression because the predictive model is more stable, and it handles the problem of correlated inputs. Regarding the disadvantages of LASSO, it can select a limited number of features and often only one feature per group of features. In addition, for low-dimensional cases, model interpretability is low [52–54]. Finally, this study only focused on in-hospital mortality, and future studies will examine the nomogram over the long term. Future studies should compare multiple nomograms using the same patient population. In addition, additional approaches could be explored for hyperglycemic patients, like thrombus aspiration combined with PCI [55].

## Conclusions

In this study, a novel nomogram was developed and is a simple and accurate tool for predicting the risk of in-hospital mortality in patients with acute STEMI who underwent primary PCI. An individualized precision prediction can be achieved for patients with acute STEMI undergoing primary PCI, thus achieving the goals of early diagnosis, early prevention, and early treatment for high-risk patients.

## Supplementary information

**Additional file 1**. Supplementary materials.

## Data Availability

The datasets used and/or analyzed during the current study are available from the corresponding author on reasonable request.

## Abbreviations

PCI: Percutaneous coronary intervention
STEMI: ST-elevated myocardial infarction
LASSO: Least absolute shrinkage and selection operator
LMCAD: Left main coronary artery disease
SDT: Symptom-to-door time
SBT: Symptom-to-balloon time
LVEF: Left ventricular ejection fraction
NSTEMI: Non-ST-elevation myocardial infarction
IABP: Intra-aortic balloon pump
PCPs: Percutaneous cardiopulmonary support
ALT: Alanine aminotransferase
AST: Aspartate aminotransferase
TG: Triglycerides
LDL-C: Low-density lipoprotein
HDL-C: High-density lipoprotein
VLDL-C: Very-low-density lipoprotein
CK-MB: Creatine kinase MB
EF: Ejection fraction
ACEI/ARB: Angiotensin-converting enzyme inhibitor/angiotensin receptor blocker
LASSO: Least absolute shrinkage and selection operator
