# Supplementary material for: Establishment and validation of a risk model for prediction of in-hospital mortality in patients with acute ST-elevation myocardial infarction after primary PCI
Source: BMC Cardiovasc Disord. 2020 Dec 9;20:513. doi: 10.1186/s12872-020-01804-7 (PMC7727168; doi:10.1186/s12872-020-01804-7)
Supplement: Supplementary file 1 — Additional file 1. Supplementary materials. [file 12872_2020_1804_MOESM1_ESM.docx]

**Supplementary Materials**

**Example of risk calculation**

For example, an elderly woman was sent to the hospital due to chest pain 2 h after onset. Wet rales could be heard in both lungs, with an area <50%. The electrocardiogram showed extensive ST-segment elevation in the anterior wall, and the heart color Doppler ultrasound showed that the ejection fraction was 45%. She was immediately given primary PCI. During the operation, we found that the lesion was at the proximal anterior descending artery, and the patient had severe thrombotic load, with a thrombus score of 5. IABP-assisted coronary interventional treatment was given. Symptom-to-balloon time was about 3 h. There was transient slow blood flow during the operation, the TIMI score was 2, and the syntax score was 25. Postoperative CK-MB was dynamically monitored; the peak appeared at 13 h after onset, and the highest level was 300 U/L. Because the patient's postoperative blood pressure was low, β-blockers and ACEI/ARB were not given.

Calculating the risk of in-hospital death of the patient:

Female, 12 points;

SDT: 120 min, 12 points;

SBT: 180 min, 12 points;

Killip classification: II, 14 points;

LMCAD: no, 0 point;

Syntax score: 25, 8 points;

Grading of thrombus: 5, 22points;

TIMI classification: II, 2 points;

Slow flow: yes, 4 points;

EF: 45%, 64 points;

Application of IABP: yes, 2 points;

Peak of CK-MB: 300U/L, 10 points;

Administration of β-blocker: no, 14 points;

ACEI/ARB: no, 12 points;

Total: 12+12+12+14+0+8+22+2+4+64+2+10+14+12=188 points, and the risk of in-hospital death was 80%.
